# A Review on ZnO Nanostructures for Optical Biosensors: Morphology, Immobilization Strategies, and Biomedical Applications

**DOI:** 10.3390/nano15211627

**Published:** 2025-10-25

**Authors:** Amauri Serrano-Lázaro, Karina Portillo-Cortez, María Beatriz de la Mora Mojica, Juan C. Durán-Álvarez

**Affiliations:** 1 Instituto de Ciencias Aplicadas y Tecnología, Universidad Nacional Autónoma de México, Circuito Exterior S/N, Coyoacán, Ciudad de México 04510, Mexico; amauri.serrano@icat.unam.mx (A.S.-L.); betarina@gmail.com (M.B.d.l.M.M.); 2 Centro de Nanociencias y Nanotecnología, Universidad Nacional Autónoma de México, Ensenada 22860, Mexico; portillo.ck90@gmail.com

**Keywords:** biosensing mechanisms, label-free detection, semiconductor nanomaterials, surface functionalization, ZnO nanostructures

## Abstract

ZnO nanostructures have attracted attention as transducer materials in optical biosensing platforms due to their wide bandgap, defect-mediated photoluminescence, high surface-to-volume ratio, and tunable morphology. This review examines how the dimensionality of ZnO nanostructures affects biosensor performance, particularly in terms of charge transport, signal transduction, and biomolecule immobilization. The synthesis approaches are discussed, highlighting how they influence crystallinity, defect density, and surface functionalization potential. The impact of immobilization strategies on sensor stability and sensitivity is also assessed. The role of ZnO in various optical detection schemes, including photoluminescence, surface plasmon resonance (SPR), localized (LSPR), fluorescence, and surface-enhanced Raman scattering (SERS), is reviewed, with emphasis on label-free and real-time detection. Representative case studies demonstrate the detection of clinically and environmentally relevant targets, such as glucose, dopamine, cancer biomarkers, and SARS-CoV-2 antigens, with limits of detection in the pico- to femtomolar range. Recent developments in ZnO-based hybrid systems and their integration into fiber-optic and microfluidic platforms are explored as scalable solutions for portable, multiplexed diagnostics. The review concludes by outlining current challenges related to reproducibility, long-term operational stability, and surface modification standardization. This work provides a framework for understanding structure–function relationships in ZnO-based biosensors and highlights future directions for their development in biomedical and environmental monitoring applications.

## 1. Introduction

**Figure 1:**
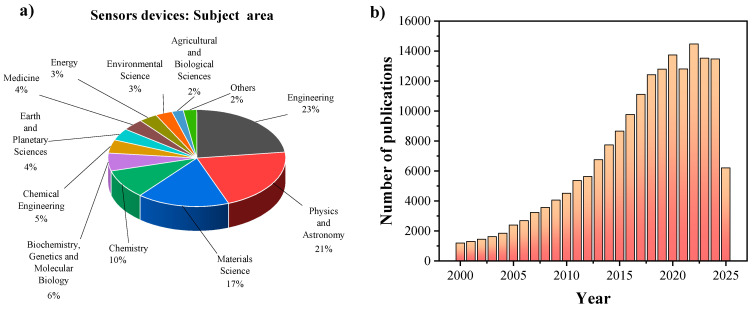
Research articles related to “Sensor/Sensors” devices categorized by (**a**) subject areas and (**b**) year of publication from 2010 to 2025 (Source: Scopus and Web of Science databases).

In recent years, the development of next-generation electronic, spintronic, optoelectronic, and sensing devices has increasingly built upon the strategic design of novel materials or the purposeful modification of existing ones, either through doping, surface decoration, or nanostructuring, to boost their overall performance. A major emphasis within the research community has been placed on uncovering correlations between material morphology and physicochemical properties (e.g., conductivity, surface area, and surface chemistry) and their influence on device functionality [1,2]. Sensing platforms play a pivotal role in modern technologies, enabling both humans and machines to keep track of and respond to chemical, mechanical, or optical stimuli, with applications spanning industrial automation, healthcare, energy, and environmental risk monitoring (Figure 1a). These systems are distinguished by their ability to enhance safety, allow real-time data acquisition, and support process optimization [3,4]. Among various sensing technologies, biosensors have attracted particular attention for their capacity to interface directly with biological systems. This growing interest is reflected in a continuous rise in research publications—an average annual growth rate of 8% since 2000, which has risen to 13% after 2013—largely brought about by advances in nanomaterial synthesis and the emergence of highly sensitive sensing platforms (Figure 1).

**Figure 2:**
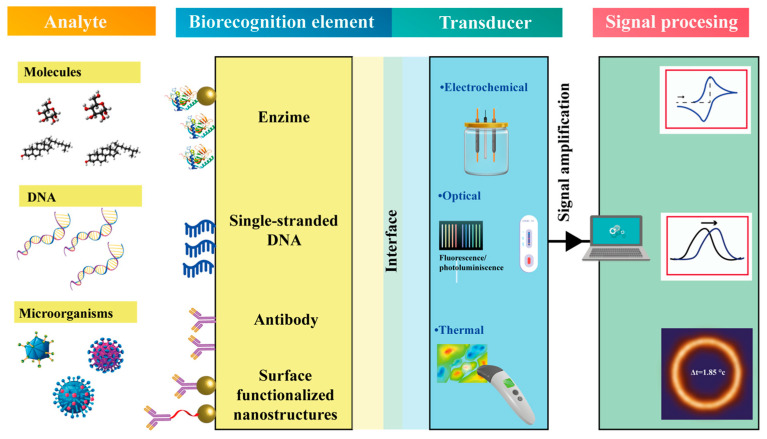
Schematic illustration of key biosensor components and sensing mechanisms for target analyte detection.

Within this broad category, biosensors have become indispensable analytical tools for detecting biological targets in environmental monitoring [5,6], food safety [7,8], and medical diagnostics [6,9]. These devices rely on bioreceptors, such as enzymes, antibodies, or DNA aptamers, to selectively identify target analytes. Upon molecular recognition, a measurable signal—chemical, electrical, optical, or thermal—is produced and quantitatively correlated with the analyte concentration (Figure 2) [9,10,11]. The selection of materials and synthesis strategies plays a decisive role, as they directly affect the availability of active sites for biomolecule immobilization, thereby influencing the overall sensitivity and selectivity of the sensing platform.

Recent progress in nanotechnology, driven by miniaturization, innovative synthesis methods, and advanced surface modification, has brought about a paradigm shift in biosensor design, enabling remarkable improvements in detection performance [11,12]. The integration of nanomaterials such as ZnO nanowires (ZnONWs) [13,14], gold nanoparticles (AuNPs) [15,16], perovskites [17,18], and graphene [19,20] has proven transformative, owing to their intrinsic nanoscale characteristics, including high surface-to-volume ratios, tunable optoelectronic properties, efficient signal amplification, and surface chemistry favorable for biomolecule immobilization and analyte detection [21,22].

The transition toward nanostructured transducers has paved the way for biosensors with superior sensitivity and selectivity, capable of detecting trace analytes in biological fluids, such as blood serum or wastewater. Semiconductors are central to these systems because of their advantageous electrical, optical, and surface properties, which are vital for signal transduction and amplification. Among them, metal oxide semiconductors, including ZnO, Fe_2_O_3_, CeO_2_, SnO_2_, and TiO_2_, stand out for their biocompatibility, non-toxicity, and catalytic activity. These benefits are complemented by their excellent electron-transfer and adsorption capabilities, making them ideal candidates for providing a suitable environment for biomolecule immobilization [11,23,24].

Among the various metal oxides, ZnO has emerged as an exceptionally versatile material for biosensing applications. Research efforts across scientific and biotechnological communities have focused on designing, synthesizing, and integrating ZnO-based nanostructures (e.g., nanoparticles, nanoflowers, and nanowires) into biosensor platforms to achieve highly sensitive and selective detection [11,24]. A comprehensive understanding of ZnO potential calls for a closer look at its intrinsic features that set it apart from other semiconductors and make it particularly suitable for these applications.

**Figure 3:**
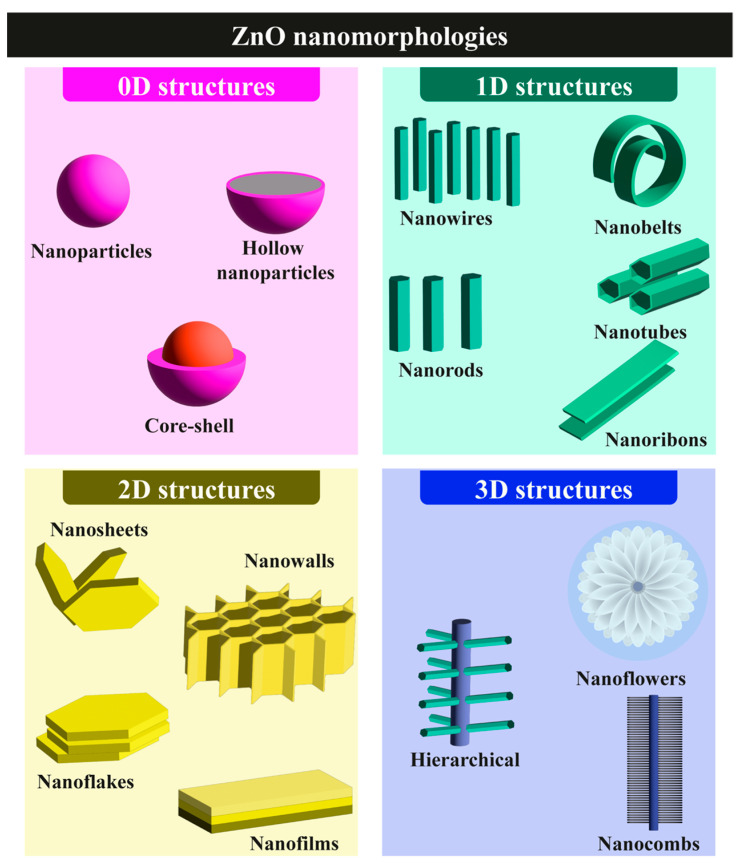
Schematic representation and classification of ZnO nanostructures: from 0D to 3D morphologies.

ZnO is an n-type II–VI semiconductor that offers several key advantages for biosensing [25]:

1. Nanostructuring flexibility: It can be fabricated through top-down (e.g., glancing-angle ion irradiation) or bottom-up (e.g., vapor-phase and chemical solution) approaches, yielding high-quality nanostructures.
2. Broad substrate compatibility: It adheres to a wide range of substrates, from flexible polymers (e.g., polyimide, polyethylene naphthalate) to rigid ceramics (e.g., alumina), and from crystalline (e.g., quartz, silicon wafers) to amorphous (e.g., glass) materials.
3. Superior physical properties: It exhibits enhanced electrical conductivity, optical transparency, and mechanical robustness compared with its bulk counterpart.
4. Morphological diversity: It can be engineered into structures ranging from zero-dimensional (0D) nanoparticles to three-dimensional (3D) hierarchical architectures, allowing tailored biosensor configurations (Figure 3).

ZnO nanostructures can be synthesized through a variety of techniques, including vapor-phase deposition, sputtering, sol–gel processing, and hydrothermal growth (see Section 2 for details). Precise control over synthesis parameters has made it possible to produce an extensive range of morphologies, like rods, tetrapods, wires, flakes, flowers, fibers, and belts [6,9,26]. This morphological versatility, combined with the distinctive physicochemical properties of ZnO, has expanded its range of applications, encompassing chemical sensors [5,27], UV photodetectors [27], smart windows [28,29], transparent electronics [29], laser and UV optoelectronic devices, solar cells, environmental remediation [14,30,31], and, notably, biosensors [19,32].

Recent studies have pointed out that ZnONWs decorated with AuNPs can substantially enhance the sensitivity of fiber-optic plasmonic biosensors, achieving detection limits as low as 0.51 pg·mL^−1^ for prostate-specific antigen [16]. Likewise, ZnONWs functionalized with DNA capture molecules have enabled the electrical detection of specific DNA sequences [33], highlighting the versatility of this material for both optical and electrochemical biosensing, as well as its applicability to a broad spectrum of biomolecules. Owing to their physicochemical properties and compatibility with a wide variety of substrates, ZnO nanostructures are increasingly regarded as promising building blocks for next-generation optoelectronic technologies and, more recently, for advanced biosensing devices. The ability to fine-tune their morphology, from individual nanoparticles to complex hierarchical architectures, has proven decisive in boosting biosensor performance.

Building upon these considerations, this review aims to shed light on the correlation between ZnO nanostructures and their physicochemical characteristics, with a particular emphasis on synthesis-dependent features and their influence on biosensing performance, especially regarding biomolecule immobilization and optical detection.

## 2. Strategies to Synthesize ZnO Nanostructures

In recent years, the performance of biosensors has been markedly improved by advances in the design of nanostructured materials, coupled with various modification strategies such as functionalization, nanoparticle decoration, and post-deposition treatments [34,35]. Modern synthesis techniques now allow precise control over nanostructure morphology, dimensions, and surface characteristics. This section examines how synthesis strategies influence the resulting material properties and, in turn, their relevance to biosensing applications.

Nanostructured ZnO used in biosensors must exhibit a high surface area, strong biomolecule-binding capability, and operational stability to achieve sub-nanomolar detection limits [6,36,37]. Chemical vapor deposition (CVD) methods are particularly well known for producing one-dimensional (1D) nanostructures, such as nanorods and nanowires, with high crystallinity, owing to the elevated temperatures (550–1000 °C) that enhance atomic mobility and give rise to well-ordered crystal lattices [13,14,38]. In contrast, hydrothermal synthesis enables the growth of 1D ZnO structures on flexible substrates at lower temperatures, making this technique compatible with polymer-based materials [39,40,41]. Thus, synthesis methodologies directly affect both the structural and functional properties of ZnO, and balancing scalability, cost, and specific device requirements remains a key design challenge.

**Figure 4:**
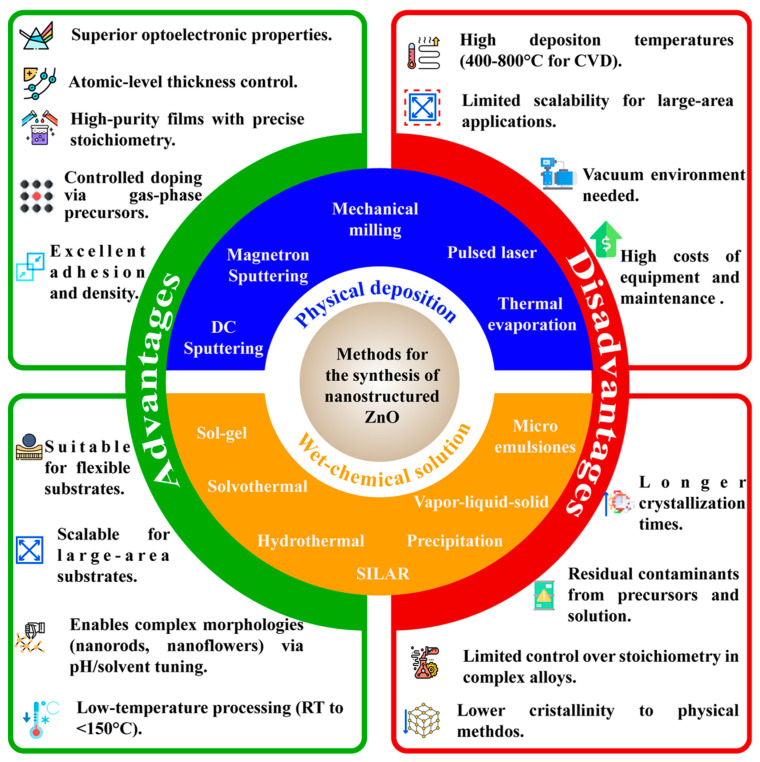
Schematic representation of the main approaches for ZnO nanostructure synthesis: physical deposition and wet-chemical solution methods, including advantages and disadvantages [25,46,47,48,49].

A variety of approaches have been assessed to synthesize ZnO nanoforms, most of which rely on bottom-up strategies that assemble fundamental building units such as atoms or molecules. These techniques are broadly classified according to how the precursor units are delivered (Figure 4).

Physical-phase methods—such as CVD and sputtering—supply atoms as gaseous precursors (e.g., carbothermal reduction of ZnO at 900–1000 °C) or as products of ablated solid targets. These approaches are highly effective for producing uniform, crystalline nanostructures but often demand energy-intensive conditions that limit large-scale implementation.

Wet-chemical methods—including hydrothermal synthesis and spray pyrolysis—involve dissolving precursors such as salts, molecular species, or precipitating agents in liquid solvents. They enable low-temperature synthesis suitable for flexible substrates; however, they call for precise control of reaction parameters to ensure reproducibility and uniformity.

Figure 4 provides a comparison between these synthesis routes and conventional fabrication techniques, bringing out the main advantages and limitations of each in terms of synthesis infrastructure, precursor types, resulting material properties, and the need for post-processing steps before device integration.

### 2.1. Physical Methods

**Chemical Vapor Deposition (CVD).** This gas-phase technique enables the controlled synthesis of thin films and nanostructures, such as nanowires and nanorods, through the thermal or plasma-assisted decomposition of volatile precursors that deposit on heated substrates [6,34]. CVD offers several versatile routes for fabricating high-performance one- and three-dimensional (1D and 3D) nanostructures, including metal–organic CVD (MOCVD), low-pressure CVD (LPCVD), and plasma-enhanced CVD (PECVD).

The synthesis of ZnO nanoforms via MOCVD [42] and PECVD [43] often encounters challenges related to substrate effects (e.g., lattice mismatch), multiple growth parameters, complex infrastructure, and the toxicity of precursors or by-products, all of which can compromise reproducibility and purity [44]. In response, LPCVD has recently gained attention for its simpler setup and improved process control. Typical operating conditions include vacuum pressures between 0.1 and 10 Torr, temperatures from 500 to 700 °C, and Ar or Ar/O_2_ gas flows of 200–400 cm^3^ min^−1^. These conditions bring about highly uniform nanostructures, which are essential for high-performance sensing devices [45].

**Vapor–Solid (VS) and Vapor–Solid–Solid (VSS).** The VS method is a catalyst-free approach used to synthesize diverse ZnO nanostructures, such as nanowires, nanotubes, nanobelts, and quasi-two-dimensional (2D) films [50,51]. Zn and O_2_ precursor vapors are transported by inert gases (e.g., Ar, He) and oxidized on heated substrates at 400–800 °C [51,52]. The substrate type can strongly influence the growth mechanism, ranging from chemically etched a-oriented sapphire to transparent conductive oxides like indium tin oxide (ITO) or fluorine-doped tin oxide (FTO) [51]. Recent improvements have made it possible to carry out VS synthesis at lower temperatures (≈580 °C) using preheating steps [53,54].

In contrast, the VSS process uses solid catalysts (e.g., AuNPs) that adsorb and diffuse precursor vapors, with crystallization occurring at the solid–solid interface. This mechanism allows for more controlled nucleation and directional growth of the resulting nanostructures.

**Vapor–Liquid–Solid (VLS).** The VLS mechanism remains the most widely used CVD-based technique for synthesizing highly crystalline 1D ZnO nanostructures [14,31,55]. It uses metal catalysts, commonly Au, Pt, Ag, or Cu, either as nanofilms (1–4 nm) or nanoparticles [13]. Growth occurs on patterned substrates or seed-layer-coated surfaces whose lattice constants closely match the growing material, thus guiding nucleation and assembly [13,29,56]. Upon heating (550–950 °C), the catalyst forms a liquid alloy with vaporized species. As the system becomes supersaturated, ZnO precipitates at the liquid–solid interface, giving rise to nanowires or nanorods [33,57].

**Pulsed Laser Deposition (PLD).** PLD uses high-energy laser pulses (e.g., KrF excimer, 248 nm) to ablate zinc or ZnO targets in an oxygen-controlled environment, depositing nanostructures onto substrates such as glass, quartz, or silicon [32,58]. Typical conditions involve 400–700 °C and 1–10 Torr of oxygen pressure. Morphology can be fine-tuned by adjusting deposition time, laser fluence, and pulse rate. When silicon (100) is used as the substrate, 1D nanostructures such as nanowires and nanoworms are obtained, whereas Al_2_O_3_ substrates yield 2D nanowalls [58].

ZnO-based composites incorporating KCN have also been developed as efficient platforms for enzyme immobilization (for instance, glucose oxidase) showing excellent biocompatibility and signal stability [59]. Despite its advantages of high purity and precise control, PLD is limited by its relatively small deposition area.

**Sputtering.** Sputtering involves bombarding a target material with high-energy Ar^+^ ions, ejecting atoms that subsequently condense on a substrate (such as glass, quartz, or silicon wafers) to form thin films or nanostructures [56,60,61]. In direct-current (DC) sputtering, an oxygen flux oxidizes Zn atoms, whereas radio-frequency (RF) sputtering uses ZnO targets to prevent charge buildup. Magnetron configurations further enhance sputtering efficiency by increasing plasma density [29,61].

Compared with PLD, sputtering supports larger deposition areas and offers greater scalability. Al-doped ZnO (AZO) films, for instance, have proven to be effective transducers for detecting analytes such as urea [61], cholesterol (25–400 mg dL^−1^) [62], and the interleukin-6 (IL-6) biomarker (0.001–100 ng mL^−1^) [63].

### 2.2. Wet-Chemical Solution Methods

Wet-chemical solution routes are among the most versatile and widely applied strategies for synthesizing ZnO nanostructures with diverse morphologies. These approaches typically rely on the reaction of zinc salt precursors (e.g., zinc acetate, nitrate, or chloride) with alkaline agents (e.g., KOH or NaOH), often in the presence of additives or structure-directing agents such as hexamethylenetetramine (HMTA) or cetyltrimethylammonium bromide (CTAB), within aqueous or mixed solvent systems. By precisely tuning synthesis parameters—including precursor concentration, pH, reaction temperature, and the nature of counterions—a broad spectrum of ZnO nanostructures can be obtained. High crystallinity and controlled dimensionality are achievable even under ambient or low-temperature conditions. The simplicity, tunable morphology, and scalability of wet-chemical methods make them particularly attractive for large-scale production.

**Hydrothermal/solvothermal.** ZnO nanomaterials can be synthesized in sealed autoclaves, where precursor species (e.g., zinc nitrate) react under controlled temperature and pressure. When water is the solvent, the process is termed hydrothermal; when non-aqueous media such as ethanol, ethylene glycol, or dimethylformamide are employed, it is known as solvothermal. Hydrothermal syntheses are typically carried out at 100–300 °C and 1–100 MPa for 2–24 h, whereas solvothermal processes often operate at lower temperatures (80–200 °C), providing enhanced morphological control due to the moderating effect of organic solvents [39,51,64].

These mild conditions, compared with CVD-based methods, make hydrothermal and solvothermal syntheses particularly suitable for flexible or temperature-sensitive substrates, including polymers [2,12] and hybrid ITO/PET platforms [65]. Furthermore, the addition of surfactants (e.g., CTAB, amines, alcohols, or glycols) and the use of seed-layered substrates significantly influence nucleation and growth mechanisms, enabling precise control of nanorod, nanosheet, or hierarchical architectures [32]. The combination of uniformity, high surface area, and scalability under relatively gentle conditions makes these routes ideal for fabricating ZnO-based flexible biosensors.

**Sol–gel method.** The sol–gel approach involves the hydrolysis and polycondensation of zinc precursors in aqueous or organic media, followed by a thermal treatment. Typically, zinc salts react with alkaline agents (e.g., NaOH or KOH) under controlled pH and temperature to yield a colloidal sol, which subsequently undergoes ageing, gelation, and calcination (400–900 °C) to produce the final oxide material [32].

For example, hydrolysis of zinc acetate dihydrate in 1 M NaOH at 50–90 °C yields ZnO nuclei that evolve into nanorods after approximately 2 h of ageing and drying. The inclusion of HMTA during hydrolysis promotes anisotropic growth, favoring the formation of ZnO nanowires [66]. Similarly, polyol-mediated routes using glycerin and isopropanol can produce nanoparticles of 50–100 nm in diameter. The calcination temperature critically affects crystallite size and aggregation, while surfactants such as CTAB promote distinctive morphologies (e.g., thorn-like nanoparticles). The use of ZnO seed layers (e.g., spray-deposited) also facilitates the vertical alignment of nanostructures [67]. Overall, the sol–gel technique offers an excellent compromise between scalability, cost efficiency, and morphological precision, making it valuable for both industrial and biomedical applications.

**Spray pyrolysis (pneumatic and ultrasonic).** Spray pyrolysis entails the atomization of a precursor solution into fine droplets that thermally decompose upon contact with a heated substrate, forming ZnO nanostructures. Two main variants are employed: pneumatic spray pyrolysis (PSP) and ultrasonic spray pyrolysis (USP) [31,68]. In PSP, droplets of 50–200 µm are generated and decomposed at substrate temperatures of 300–500 °C, whereas USP produces much finer droplets (1–50 µm) through high-frequency vibrations, allowing efficient decomposition at lower temperatures (200–450 °C) [32].

These milder temperature conditions enable deposition on flexible substrates [69]. Both PSP and USP are compatible with doping strategies; for instance, the preparation of AZO films by incorporating dopant precursors into the spraying solution. However, USP generally offers superior morphological uniformity and enhanced optoelectronic properties. Consequently, spray pyrolysis represents a scalable, straightforward, and cost-effective synthesis method, well aligned with the demands of biosensing applications [70].

Overall, wet-chemical synthesis routes enable the preparation of ZnO nanostructures with 0D, 1D, 2D, and 3D morphologies, each possessing distinct advantages. By fine-tuning synthesis parameters, it is even possible to obtain multiple morphologies from a single method. Choosing an appropriate route should therefore be guided by the intended application; for instance, wearable biosensors benefit from low-temperature, hydrothermal-based syntheses that offer excellent scalability and substrate compatibility. The following sections address how specific ZnO morphologies are integrated into biosensors and how their structural and physicochemical characteristics influence their performance as transducer materials.

### 2.3. ZnO Nanosystems: A Stable Platform for Biosensing Applications

The growing incorporation of ZnO nanosystems into biosensing technologies arises from their exceptional ability to provide stable and biocompatible platforms for biomolecule immobilization while preserving biological activity. Their high surface reactivity, strong adsorption capacity, and intrinsic catalytic efficiency make ZnO nanostructures particularly well suited for the fabrication of biosensors with enhanced analytical performance. Each morphological class, ranging from zero-dimensional (0D) to three-dimensional (3D) architectures, offers distinct advantages that affect critical aspects of biosensor operation, including biomolecule immobilization strategies, analyte recognition modes, and signal transduction mechanisms.

**Figure 5:**
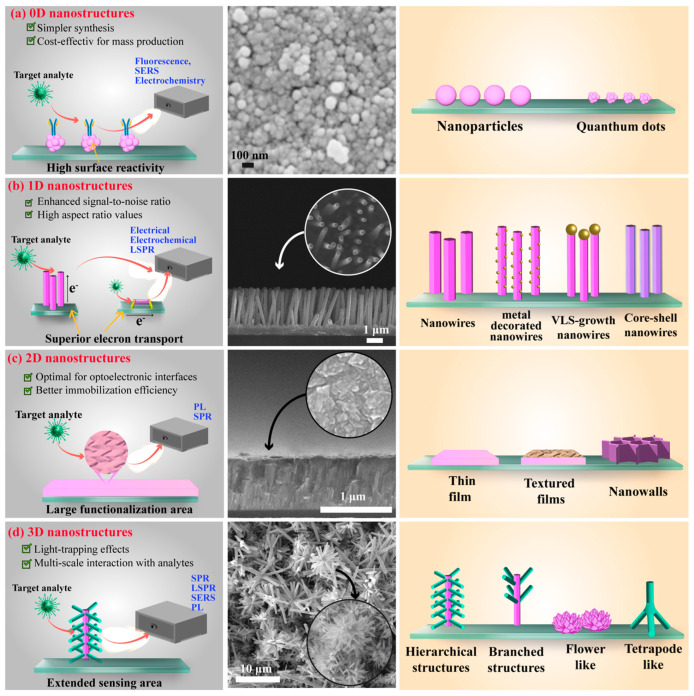
Schematic representation of ZnO nanosystem integration in biosensing devices: (**a**) 0D, (**b**) 1D, (**c**) 2D, and (**d**) 3D. SEM and TEM micrographs are original and were obtained by the authors from experiments involving the synthesis of ZnO nanostructures.

Figure 5 presents a comparative overview of the main ZnO nanostructure types (0D, 1D, 2D, and 3D), displaying representative SEM micrographs along with their corresponding synthesis advantages and functional benefits for sensing applications. A concise yet comprehensive discussion of the main physicochemical features and typical uses associated with each morphology is provided, emphasizing how dimensionality and structural design influence the performance of ZnO nanomaterials in biosensing systems.

#### 2.3.1. 0D ZnO Nanostructures

Zero-dimensional (0D) ZnO nanosystems, such as quantum dots (QDs) and nanoparticles, are defined by the confinement of all three spatial dimensions within the nanoscale range (1–100 nm). These structures can be synthesized by various wet-chemical approaches, including hydrothermal, microwave-assisted, sol–gel, and co-precipitation methods. Owing to their nanoscale dimensions, which are comparable to those of many biomolecules, 0D ZnO nanostructures exhibit enhanced surface interactions that provide an optimal environment for biomolecule adsorption and lead to significant amplification of optical and electronic signals.

In biosensing applications, their high surface area (Figure 5a) enables efficient biomolecule immobilization and promotes strong interactions with target analytes. Furthermore, their large surface-to-volume ratio markedly enhances sensitivity, making them excellent candidates for ultrasensitive biosensors [11]. ZnO QDs have been effectively incorporated into biosensing systems by exploiting fluorescence modulation mechanisms such as Förster Resonance Energy Transfer (FRET) and electron transfer. For instance, Zhao et al. synthesized 3–5 nm ZnO QDs capped with 3-aminopropyltriethoxysilane (APTES) for dopamine detection. Fluorescence quenching enabled quantitative detection across a linear range of 0.05–10 µM, with a detection limit of 12 nM. The quenching phenomenon was attributed to electron transfer from the ZnO QDs to oxidized dopamine-quinone species [36]. Similarly, Gu et al. used ZnO QDs as fluorescent and electrochemical labels for detecting the carbohydrate antigen 19-9 (CA 19-9), a recognized biomarker for pancreatic cancer. The biosensor exhibited a dynamic electrochemical response from 0.1 to 180 U mL^−1^ with a detection limit of 0.04 U mL^−1^. Its optical photoluminescence response was linear between 1 and 180 U mL^−1^, achieving a detection limit of 0.25 U mL^−1^. The remarkable analytical performance was ascribed to the small and uniform size of the ZnO QDs (average ≈ 4.8 nm) and their favorable surface chemistry, which facilitated efficient biomolecule immobilization [71].

#### 2.3.2. One-Dimensional ZnO Nanostructures

One-dimensional (1D) ZnO nanostructures are characterized by two dimensions confined within the nanoscale range (1–100 nm), while the third extends into the micrometer scale. Representative examples include nanowires, nanorods, nanotubes, and nanobelts, which can be synthesized through both gas-phase techniques (e.g., vapor–liquid–solid and vapor–solid mechanisms) and liquid-phase routes (e.g., sol–gel, electrochemical deposition, solvothermal, and hydrothermal processes). When properly synthesized, these 1D nanostructures display distinctive physicochemical features—such as quantum confinement in individual nanowires or nanorods—that render them particularly useful for applications in light-emitting diodes, photocatalysis, and sensing [25].

In biosensing, 1D ZnO nanomaterials are highly valued for their high aspect ratio and large surface-to-volume ratio, which enable efficient immobilization and loading of enzymes and other biomolecules. Their elongated geometry also provides a direct and stable pathway for charge-carrier transport (Figure 5b), facilitating rapid electron transfer between enzyme redox centers and electrode surfaces. These properties make 1D ZnO nanostructures candidates for developing high-performance, robust biosensor platforms [11,24].

Sarangi et al. investigated hydrothermally synthesized ZnO nanorods for glucose detection within the concentration range of 9–540 mg dL^−1^ using photoluminescence analysis. The nanorods exhibited catalytic activity comparable to that of oxidase enzymes typically used in conventional enzymatic glucose sensors. Moreover, they demonstrated superior crystallinity and a stronger near-band-edge photoluminescence (PL) intensity than bulk ZnO, confirming their excellent optical quality [72]. In another study, Gu et al. fabricated ZnO nanorods with an average diameter of 80 nm for the detection of phenol. Their high crystallinity, and well-defined surface favored biomolecule immobilization and facilitated efficient electron transfer during sensing. Electrochemical analysis revealed that the biosensor achieved a sensitivity of 103.08 µA mM^−1^ at phenol concentrations above 20 µM and 40.76 µA mM^−1^ at concentrations below 20 µM [73].

#### 2.3.3. Two-Dimensional ZnO Nanostructures

Two-dimensional (2D) ZnO nanostructures are defined by having one dimension confined to the nanoscale while the other two extend laterally on the micrometer scale. Typical morphologies include nanosheets, nanoplates, nanowalls, nanoflakes, and nanodisks. These architectures can be fabricated through both top-down and bottom-up strategies. Bottom-up approaches, such as CVD and wet-chemical synthesis, generally produce materials with fewer defects and more uniform chemical composition due to the precise control of reaction conditions. Conversely, top-down methods rely on the physical transformation of bulk ZnO into thin layers by means of mechanical or liquid exfoliation, or laser ablation.

2D ZnO nanomaterials exhibit excellent charge transport and surface properties, making them suitable for diverse applications, including sensors, piezoelectric devices, supercapacitors, and photocatalysis [48]. In biosensing, 2D structures provide large, well-defined surfaces for biomolecule immobilization (Figure 5c) and enable the simultaneous detection of multiple analytes [24]. For instance, Iyer et al. used ultrasonic spray pyrolysis to deposit ZnO thin films for the detection of dengue virus DNA. The films exhibited high surface area and catalytic efficiency, while their isoelectric point favored DNA immobilization via electrostatic interactions. Their findings confirmed the strong electrostatic attraction and high adsorption capacity of ZnO films toward DNA molecules [74]. Similarly, Kaur et al. developed a biosensor based on a ~200 nm thick ZnO film deposited by radio-frequency (RF) sputtering onto gold-coated glass prisms for the detection of *Neisseria meningitidis* DNA. Using surface plasmon resonance (SPR) as the transduction mechanism, the biosensor achieved a detection limit of 5 ng μL^−1^ across a linear range of 10–180 ng μL^−1^. The enhanced sensitivity was attributed to the porous, nanostructured morphology of the ZnO films, which provided efficient biomolecule loading and improved analyte–biomolecule interactions [75].

#### 2.3.4. Three-Dimensional ZnO Nanostructures

Three-dimensional (3D) ZnO nanostructures consist of self-assembled building blocks forming complex hierarchical architectures. These structures typically display high porosity, large surface area, and elevated aspect ratios, often surpassing those of 1D counterparts. Such features are particularly beneficial for applications requiring enhanced charge-carrier mobility and improved light absorption (Figure 5d). In biosensing, the high density of reactive sites in 3D architectures facilitates extensive biomolecule immobilization, thereby enhancing both the sensitivity and overall efficiency of the device [11].

Lei et al. synthesized ZnO nanotetrapods (d ≈ 200 nm, l = 2–3 µm) for quantitative glucose detection. The resulting biosensor exhibited a low detection limit of 4 µM and high sensitivity (25 µA mM^−1^ cm^−2^) over a linear glucose concentration range of 0.005–6.5 mM. The outstanding performance was attributed to the multiterminal charge conduction of the tetrapod network and its large specific surface area [76]. In another study, Qurashi et al. prepared ZnO nanotetrapods using a novel microwave-assisted approach for the detection of bisphenol A. The biosensor achieved a detection limit of 1.5 nM and a sensitivity of 5.0 µA nM^−1^ cm^−2^ within a linear range of 12.4 nM–1.2 µM. The enhanced response was attributed to the high surface area and efficient electron-transfer properties of the 3D ZnO framework [77].

The broad diversity of ZnO morphologies—from 0D quantum dots to 3D hierarchical networks—enables precise tuning of biosensor performance through structural control. Critical factors such as surface area, charge-transport pathways, and active-site density directly influence key analytical parameters, including sensitivity, detection limit, and signal amplification. As demonstrated by the examples above, 0D and 3D nanostructures are particularly effective in maximizing surface interactions and biomolecule immobilization, whereas 1D and 2D morphologies excel in facilitating charge transport and enabling selective analyte detection. Consequently, the selection of an appropriate ZnO morphology must be carefully aligned with the intended biosensing modality, whether electrical, electrochemical, or optical.

In the following section, the discussion will focus on how biomolecule immobilization strategies can be optimized to harness the unique advantages of each ZnO morphology, thereby further enhancing the overall performance of ZnO-based biosensors.

## 3. Functionalization Strategies of ZnO Nanostructures

As shown in Figure 2, biosensing devices operate through several detection mechanisms (electrical, thermal, and optical) to identify specific analytes and generate measurable signals. Beyond the fabrication of nanostructured transducers, the immobilization of biomolecules represents a decisive step in developing high-performance biosensors. For optimal operation, the biomolecule serving as the functionalization agent must display strong adhesion to the transducer surface, as well as high structural stability and molecular selectivity.

**Figure 6:**
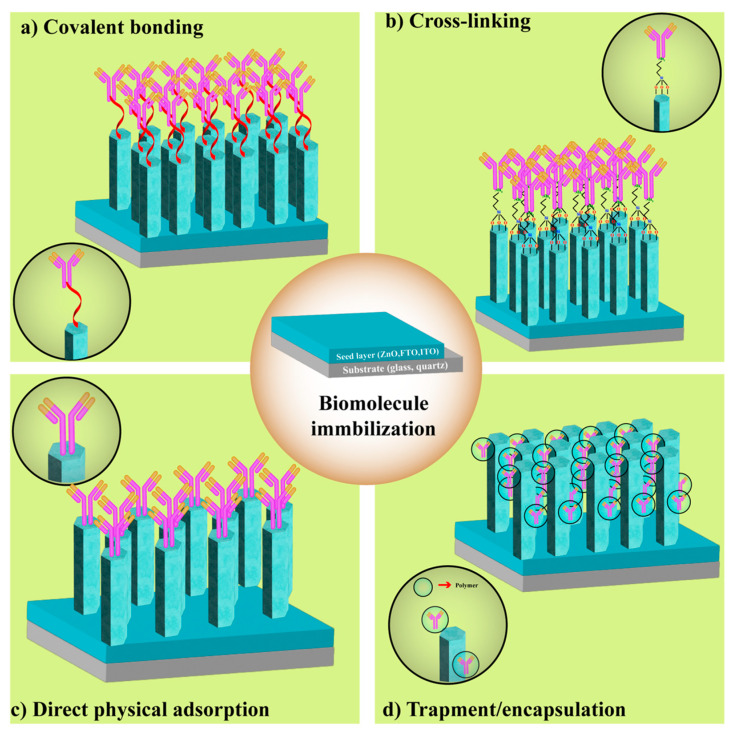
Schematic representation of immobilization strategies for target biomolecules on 1D ZnO nanostructures: (**a**) covalent bonding, (**b**) cross-linking, (**c**) direct physical adsorption, and (**d**) entrapment/encapsulation approaches.

Figure 6 illustrates the most common immobilization strategies on nanostructured platforms, including covalent attachment, physical adsorption, entrapment, and cross-linking.

Table 1 summarizes the advantages, limitations, and typical application areas of each approach in the biosensing context. Together, these resources provide essential criteria for selecting the most appropriate strategy to build up a bioselective layer on ZnO-based nanosystems.

ZnO nanostructures offer tunable surface chemistry, enabling the implementation of diverse immobilization routes that have been extensively investigated for anchoring a broad range of biomolecules. These include low-molecular-weight compounds—such as creatinine (113 Da), formaldehyde (30 Da), and formate (45 Da)—as well as proteins, antibodies, nucleic acids (e.g., DNA), and even whole cells [9,80]. In covalent immobilization, hydroxyl-rich ZnO surfaces can form stable bonds with antibodies through silanization agents, such as APTES (3-aminopropyltriethoxysilane), making them suitable for implantable glucose sensors and related biomedical applications.

In the case of physical adsorption, the high isoelectric point of ZnO (~9.5) promotes strong electrostatic interactions with negatively charged biomolecules, such as DNA strands used for pathogen detection (e.g., Salmonella, *Bacillus cereus*, SARS-CoV-2). Enzymatic biosensors, on the other hand, benefit from three-dimensional (3D) porous ZnO architectures (e.g., such as nanoflowers) that can encapsulate and protect enzymes, preserving their catalytic activity. Moreover, the use of cross-linking agents contributes to stabilizing multi-enzyme systems, facilitating multiplexed detection.

The efficiency of biomolecule immobilization on ZnO nanostructures primarily depends on two critical factors [21]:

1. **Surface chemistry.** Electrostatic interactions, surface area, loading capacity, and the number of active binding sites are governed by surface charge, porosity, crystalline orientation, and surface functional groups.
2. **Immobilization strategy.** This encompasses covalent conjugation via silane agents (e.g., APTES), physical adsorption, electrochemical deposition, and other surface modification techniques.

The chosen immobilization method not only determines the stability and functional integrity of the biomolecule but also gives rise to significant variations in biosensor performance under different operating conditions. Table 2 provides a comprehensive overview of how these strategies have been applied across various ZnO morphologies and biosensing platforms. Taken together, this analysis offers a solid foundation for selecting the most appropriate immobilization approach, which will be further discussed in the following sections in relation to overall sensor performance.

### 3.1. Covalent Binding Methods

Covalent immobilization involves the formation of stable chemical bonds between functional groups in biomolecules and activated ZnO surfaces. Typical reactive moieties include amines (–NH_2_), carboxylic acids (–COOH), hydroxyls (–OH), phosphates, aldehydes (–CHO), and thiols (–SH), which may react directly or with the assistance of cross-linking agents. Two main approaches are commonly used: the direct method and cross-linker-assisted techniques.

In direct immobilization, the intrinsic or post-treated surface chemistry of ZnO nanostructures enables the straightforward attachment of biomolecules (Figure 6a). For instance, ZnO nanoparticles synthesized via green routes using plant extracts, such as *Artocarpus heterophyllus*, *Carom–Trachyspermum ammi*, or *Nyctanthes arbor-tristis*, naturally bear surface-bound functional groups (including aldehydes, amines, and terpenoids) that can act as biotemplates [93,94]. These surface groups form Schiff base linkages (–N=CH–) with primary amines in proteins, such as antibodies, thereby allowing covalent immobilization without the need for additional cross-linking agents [94]. Another direct approach involves activating ZnO surfaces through plasma or hydrogen peroxide treatment, which gives rise to the formation of surface peroxides (ZnO_2_). These reactive species can subsequently bond covalently with thiol or amine groups in enzymes, like glucose oxidase, thus enabling stable immobilization through radical-based reactions while bypassing the use of external cross-linkers [95,96].

Cross-linker-assisted strategies, on the other hand, have been extensively used to enhance covalent biomolecule attachment on ZnO nanostructures (Figure 6b). Among them, glutaraldehyde (GA) remains one of the most widely applied cross-linkers, forming Schiff base bonds between the ZnO surface and biomolecule functional groups. For example, trypsin has been successfully immobilized onto chitosan-coated ZnO nanoparticles using GA as a linker [97]. In another case, a combination of GA and Nafion were used to immobilize glucose oxidase on ZnO-nanorod/Au electrodes, improving biosensor performance by preventing enzyme leaching and maintaining mechanical stability during operation [98].

Silanization approaches, using APTES and mercaptopropyltrimethoxysilane (MPTMS), have also proven effective for biomolecule immobilization on ZnO surfaces. Tiwari et al. [80] developed a ZnO-based paper biosensor using hydrothermally synthesized 1D nanorods functionalized with antibodies through APTES, enabling amide (–CONH_2_) bond formation. This device was designed to detect myoglobin, a cardiac biomarker, and exhibited excellent selectivity even in complex biological matrices, achieving a threefold increase in sensitivity compared to conventional paper-based ELISA methods. Furthermore, surface modification with 3-mercaptopropionic acid (MPA) provides carboxyl-terminated ZnO surfaces suitable for EDC/NHS-mediated coupling reactions in biomedical applications [99]. Han et al. [100] reported a CdSe–ZnO flower-rod core–shell biosensor for norovirus RNA detection, in which MPA enabled the covalent attachment of DNA probes via the coupling of probe –NH_2_ groups with surface carboxyl moieties. The resulting biosensor achieved an improved current response of up to 0.1 mA and demonstrated high sensitivity within the 0–5.10 nM concentration range, with a detection limit of 0.50 nM.

Taken together, these strategies build upon the versatile surface chemistry of ZnO nanostructures, offering robust and tunable platforms for covalent biomolecule immobilization, which ultimately enhances biosensor stability, selectivity, and analytical performance.

### 3.2. Direct Physical Adsorption Methods

In contrast to covalent approaches, physical adsorption relies on a simpler mechanism in which biomolecules are immobilized on ZnO surfaces through non-covalent interactions, such as electrostatic forces, van der Waals interactions, and hydrogen bonding (Figure 6c) [101]. The typical procedure involves immersing the ZnO-based substrate into a biomolecule-containing solution, followed by incubation and rinsing steps. The main advantages of this technique lie in its cost-effectiveness and the preservation of biomolecular activity throughout the immobilization process [35,79,101].

Physical adsorption has been successfully carried out on a wide range of platforms, including membranes (e.g., nitrocellulose), hydrophobic surfaces (e.g., polymer-coated substrates), and ZnO-based nanomaterials [35]. Despite its simplicity, however, this method is limited by the potential desorption of biomolecules due to the inherently weak and reversible nature of the non-covalent interactions. This drawback may lead to reduced biosensor stability and sensitivity, as well as to possible contamination issues. Factors such as pH, temperature, biomolecule concentration, and the physicochemical properties of the surrounding medium strongly influence adsorption efficiency and reproducibility.

Several studies have investigated the electrostatic interactions between ZnO nanostructures, such as nanoparticles, nanorods, and nanosheets, and proteins including bovine serum albumin (BSA) and fibrinogen (Fg). It was observed that the zeta potential of ZnO nanostructures became increasingly negative upon immersion in a buffer solution (pH 7–7.3) containing BSA. Thermodynamic analyses revealed negative enthalpy and entropy changes, bringing about strong evidence that hydrogen bonding and van der Waals interactions dominate the adsorption process [21]. Compared with Fg, BSA exhibited higher adsorption efficiency across ZnO morphologies, likely due to its lower isoelectric point (4.5 for BSA vs. 5.5 for Fg) and smaller molecular weight (4.5 kDa vs. 5.5 kDa). These findings highlight how the intrinsic physicochemical properties of biomolecules govern their immobilization efficiency on ZnO surfaces.

In another study, anti-immunoglobulin G (anti-IgG) antibodies conjugated with AuNPs were immobilized via direct adsorption onto ZnONWs synthesized through wet-chemical routes. The resulting biosensor exhibited a linear correlation between the piezoelectric response and anti-IgG concentration within the range of 10^−7^–10^−3^ g mL^−1^, achieving a limit of detection (LOD) of 6.9 ng mL^−1^ [102].

### 3.3. Entrapment-Encapsulation Strategies

Biomolecule immobilization through entrapment or encapsulation enhances biosensor performance by increasing the effective surface area of the immobilization matrix. In this approach, biomolecules are physically confined within a sol–gel or polymeric network, such as chitosan, Nafion, resins, or surfactants. rather than being directly attached to the nanostructured substrate (Figure 6d). Although the biomolecules are trapped within the matrix, the porous nature of these materials allows analytes to diffuse freely and interact with the biorecognition elements, thereby maintaining sensor responsiveness. This strategy typically brings about enhanced sensitivity, yet it can face certain limitations, like nonspecific adsorption and reduced long-term stability [10,79].

In recent years, nanostructured inorganic–organic hybrid materials have drawn significant attention as promising architectures for biosensor fabrication. Organic matrices such as Nafion and chitosan, as well as conductive polymer hydrogels—including polyaniline (PAni) and poly(3,4-ethylenedioxythiophene) (PEDOT)—are widely used. These materials provide a biocompatible microenvironment that closely mimics physiological conditions, facilitating biomolecule entrapment while preserving their structural conformation and catalytic activity. Due to their high hydrophilicity, large surface area, and excellent biocompatibility, such hydrogels stabilize biomolecules effectively and greatly extend their operational lifetime [10,79,101]. In this regard, Khazaei et al. developed an impedimetric glucose biosensor based on a 3D nanoporous ZnO/chitosan composite, in which polyvinyl alcohol (PVA) served as a sacrificial polymer to generate a uniform ZnO framework. The resulting sensor exhibited a linear detection range of 1.0–18.0 mM with a LOD value of 0.2 mM. The improved performance was attributed to the hydroxyl- and amine-rich functional groups of chitosan, as well as to the favorable isoelectric point difference between the ZnO/chitosan platform (~9.0) and the glucose oxidase enzyme (~4.2), which gave rise to strong and stable enzyme immobilization [103]. Similarly, Yen et al. reported a nanocomposite consisting of ZnO nanorods with poly(3,4-ethylenedioxythiophene) doped with polystyrene sulfonate (ZnO/PEDOT:PSS) for the ultrasensitive detection of Pb^2+^ ions in human serum. The device showed a linear response in the concentration range of 0.005–10 ppm, achieving a detection limit of 12 ppb in both phosphate-buffered saline (PBS) and commercial serum samples [104].

Collectively, these studies build upon the concept that entrapment-based strategies provide a versatile route to integrate biomolecules within flexible, biocompatible, and highly porous networks. However, optimizing matrix composition and controlling nonspecific binding remain essential challenges to further advance the stability and reproducibility of ZnO-based biosensing systems.

## 4. Optical Detection of Biomolecules in ZnO-Based Platforms

**Figure 7:**
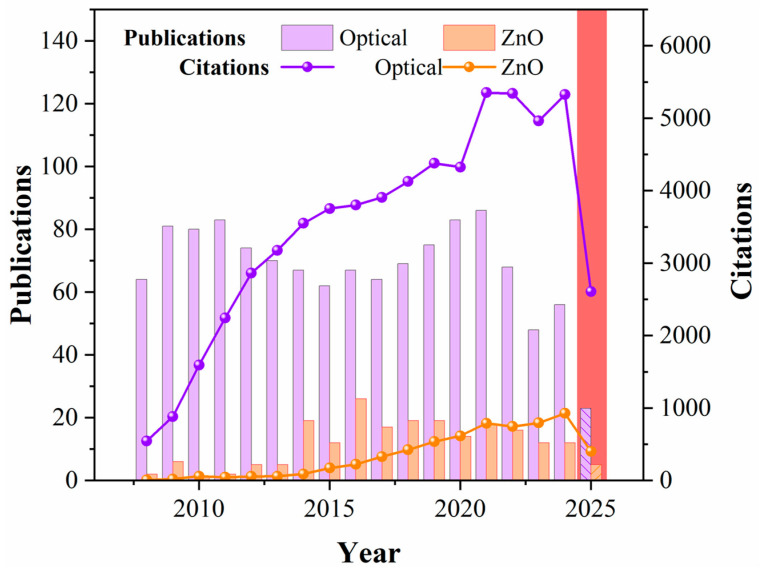
Bibliometric analysis of publications and citations related to biosensors using keyword-based queries: (1) “biosensors” + “optical” (purple) and (2) “biosensors” + “ZnO” (orange). The area in red highlights the numbers in current year. Data obtained from SCOPUS and Web of Science (accessed on 22 June 2025).

As previously discussed, the unique optical and electronic properties of nanostructured ZnO support the development of next-generation biosensing technologies by enabling the sensitive and selective detection of specific targets. A bibliometric analysis provides valuable insight into the evolution and scientific impact of research in this domain. Figure 7 presents a comparative overview of publication and citation trends in optical biosensing and ZnO-based biosensor research.

The results indicate that optical biosensing constitutes a well-established and highly cited field, with more than 5000 citations in recent years. In contrast, ZnO-based biosensing is emerging as a rapidly expanding niche within the broader area of optical detection, reflecting its growing scientific and technological relevance.

At the nanoscale, a clear relationship can be drawn between synthesis routes, surface reactivity, and morphology, factors that collectively dictate the optimization of optical signal transduction. Consequently, biomolecule immobilization strategies play a decisive role in determining the sensitivity, selectivity, and stability of ZnO-based optical biosensors. The tunable physicochemical characteristics of ZnO nanostructures give rise to a variety of advanced optical detection techniques, including:

- Surface plasmon resonance (SPR) and localized SPR (LSPR);
- Fluorescence-based;
- Surface-enhanced Raman scattering (SERS);
- Photoluminescence (PL) spectroscopy biosensors.

**Figure 8:**
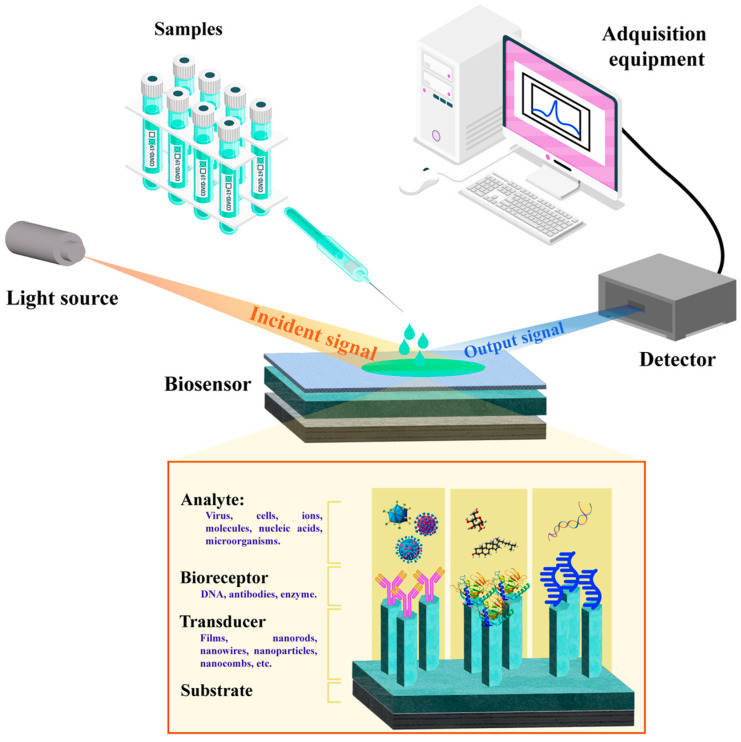
Schematic illustration of an optical biosensor showing the interaction between ZnO nanostructures (transducer), a bioselective layer, and the target analyte.

This section builds upon the foundational concepts of ZnO nanostructure synthesis, functionalization, and sensing mechanisms to elucidate how both intrinsic and defect-related optical responses can be harnessed for highly sensitive, often label-free, biomolecule detection. Optical biosensing stands out due to its capacity to convert biological or chemical recognition events into quantifiable optical signals, frequently in real time and even in complex matrices [27,105]. This functionality arises from the precise modulation and analysis of light–matter interactions, as illustrated schematically in Figure 8. Moreover, the morphology, surface chemistry, and defect density of ZnO nanostructures can be deliberately engineered to bring about substantial enhancements in optical sensitivity and selectivity [37,106].

As previously noted, controlling the dimensionality of ZnO nanostructures exerts a deep influence on biosensor sensitivity, selectivity, and overall performance. For instance, 0D nanostructures exhibit exceptionally high surface-to-volume ratios and size-dependent fluorescence, making them ideal for fluorescence-based and SERS biosensors, particularly when integrated with plasmonic metals. One-dimensional structures, by contrast, display remarkable light-trapping and anisotropic charge-transport properties, which bring about enhanced photoluminescence and surface plasmon resonance responses, enabling lower detection limits for targets such as DNA. Two-dimensional nanosheets provide extensive, planar surfaces that allow dense bioreceptor immobilization while minimizing aggregation, thereby improving operational stability and sensor longevity. Meanwhile, 3D hierarchical architectures, such as ZnO nanoflowers, facilitate analyte diffusion and accessibility, reducing nonspecific binding and improving both selectivity and reproducibility.

A wealth of experimental and theoretical evidence highlights the pivotal role of ZnO morphology in determining biosensor performance. This morphological control is thus regarded as a key design parameter in the development of next-generation optical biosensors. In general, optical biosensing systems can be divided into two main categories:

1. Label-free systems, such as SPR, which allow direct detection of analytes without additional tags.
2. Labeled systems, which rely on fluorescence or chemiluminescence tags to amplify the detection signal.

Table 3 provides a summary of key optical biosensing techniques, their underlying principles, and main advantages and limitations. The integration of nanostructured ZnO improves biosensor performance through multiple mechanisms, including (a) increased surface area for biomolecule immobilization, (b) enhanced charge transfer efficiency, and (c) intrinsic PL and UV absorption properties. Collectively, these features set ZnO-based materials apart as highly versatile components in PL-, SPR-, and SERS-based biosensing platforms, enabling the ultrasensitive detection of proteins, nucleic acids, pathogens, and low-molecular-weight analytes.

### 4.1. Surface Plasmon Resonance (SPR) and Localized-SPR (LSPR) Biosensors

SPR and LSPR are label-free optical sensing techniques that exploit the interaction between light and metallic surfaces to detect molecular binding events in real time. Both rely on variations in the local refractive index caused by biomolecular interactions; however, they differ in their underlying physical mechanisms and experimental configurations [16,21].

In SPR, analyte binding events at the metal–dielectric interface induce measurable shifts in the resonance angle or wavelength, enabling real-time, label-free analysis. LSPR, in contrast, uses metallic nanoparticles that confine the plasmonic field locally, leading to enhanced sensitivity and multiplex detection capabilities. Gold and silver nanostructures are typically employed due to their strong plasmonic absorption in the visible region. The incorporation of ZnO into metallic nanoparticle systems modifies their structural, electronic, and optical characteristics, giving rise to hybrid nanocomposites with improved biosensing performance [34,111]. In this context, Włodarski et al. [111] demonstrated, through PL and optical transmittance analyses, that defect density in ZnO nanotubes and the size distribution of metal nanoparticles critically affect plasmonic coupling at the metal/ZnO interface. Their results revealed that Ag/ZnO systems enhance UV emission via defect-level–LSPR (DL-LSPR) coupling, whereas Au/ZnO composites exhibit PL quenching, likely due to ohmic contact formation between ZnO and Au. Chou et al. [15] further observed a redshift in the SPR peak from 524 nm in air to 534.5, 535.5, and 539 nm in water, ethanol, and methanol, respectively, confirming the sensitivity of metal/ZnO systems to refractive index variations in diverse environments.

**Figure 9:**
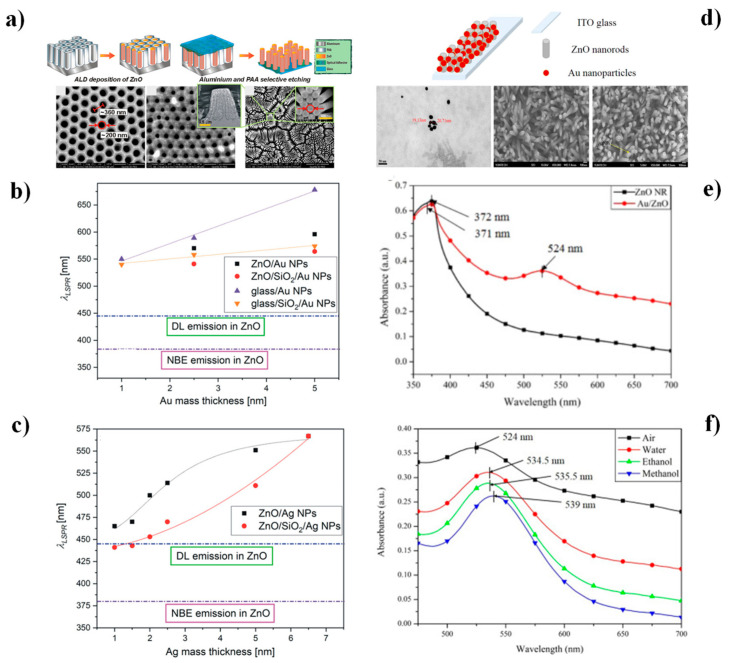
(**a**) Preparation methodology of ZnO NTs on a porous anodic alumina (PAA) template on an Al substrate and plasmonic resonance (λLSPR) as a function of (**b**) Au and (**c**) Ag film mass thickness (adapted from [111]). (**d**) Configuration of an Au-modified ZnO film for the growth of ZnO nanorods (NRs) and its absorbance spectra (**e**) before and (**f**) after exposure to different environments (adapted from [15]).

At the metal–ZnO interface, the formation of Schottky barriers promotes charge transfer from the ZnO conduction band to the Au conduction band, thereby enhancing the interaction of free electrons with target biomolecules in LSPR-based sensors [20,111]. In this context, Kim et al. [16] developed a 3D nanostructured sensing platform by decorating ZnONWs with AuNPs, thereby increasing the active surface area and improving light-trapping efficiency to amplify LSPR sensitivity (Figure 9a–c). Using a fiber-optic (FO)-based configuration, they successfully detected prostate-specific antigen (PSA), typically present at ng L^−1^ levels in young individuals. While systems containing only AuNPs achieved detection within the 0.01 pg mL^−1^ ng·mL^−1^ range (LOD: 2.06 pg mL^−1^), the 3D Au/ZnONWs extended the dynamic range from 0.001 pg mL^−1^ to 1 ng mL^−1^, achieving a lower LOD of 0.51 pg mL^−1^. Subsequent studies have built upon this approach by modifying AuNP/ZnO systems with Nafion, a cation-exchange polymer known for protecting enzymatic activity and blocking interferents (molecules structurally similar to the target analyte, such as ascorbic acid in uric acid sensors) thereby improving recognition selectivity [112]. For example, Cheng et al. [107] introduced an SPR-based dual-analyte platform that integrated ZnO nanoparticles with a thin Au film and Nafion encapsulation to simultaneously detect urea and uric acid (Figure 9d–f). Nafion acted as a selective barrier, spatially isolating the ZnO–urease and ZnO–uricase sensing regions, enabling parallel detection. This system achieved sensitivities of 1.6 nm mM^−1^ for urea (range: 1–9 mM) and 36 nm mM^−1^ for uric acid (range: 0.05–0.5 mM).

### 4.2. Fluorescence-Based Biosensors

Fluorescence-based biosensors represent a powerful analytical approach for detecting light-emitting compounds, whether through intrinsic fluorescence or via labeled analytes. By precisely selecting synthesis methods, ZnO nanoarchitectures can be engineered with tailored morphologies, optimized crystallinity, and controlled defect densities to meet the optical requirements of biosensing. These include minimizing autofluorescence and avoiding spectral overlapping with common fluorophores, both essential for achieving a high signal-to-noise ratio.

Surface-enhanced fluorescence (SEF) strategies, which use nanostructured materials as hosts for fluorophores or intrinsic emitters, have proven particularly effective in amplifying detection signals. In ZnO-based platforms, enhancement processes generally outweigh quenching effects, enabling highly sensitive fluorescence readouts. Moreover, owing to its broadband emission enhancement across diverse morphologies—from 0D nanoparticles to 1D rods and 3D flower-like assemblies—ZnO also lends itself to multiplexed detection schemes [34].

**Figure 10:**
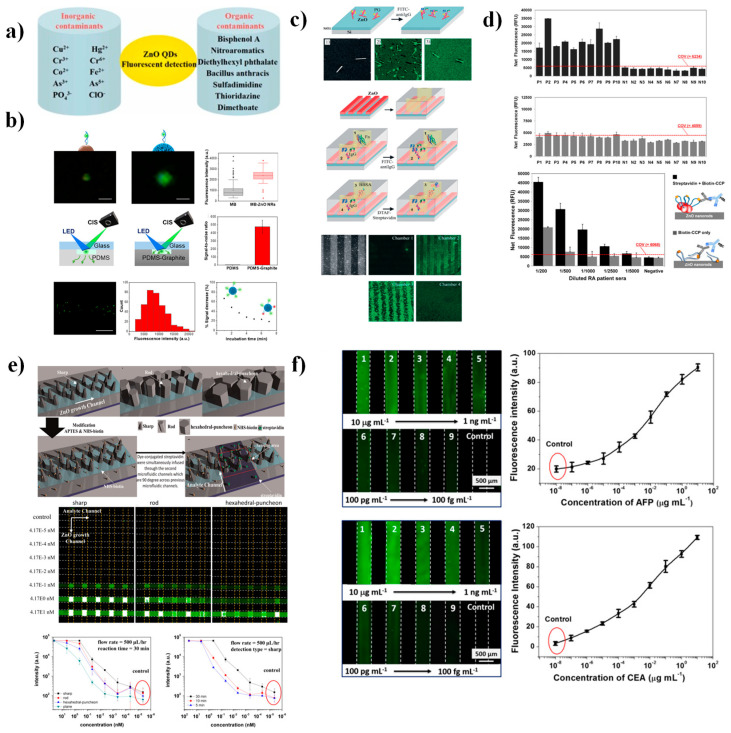
Fluorescence-based applications of ZnO nanostructures. (**a**) detection of environmental contaminants via ZnO QDs [113]. Biosensing strategies for (**b**) biomarker sensing in cTnI aptamer-functionalized ZnO NRs [108], (**c**) protein G-functionalized ZnO nanoplatforms for detecting protein–protein interactions (biotinylated bovine serum albumin and DTAF-streptavidin) [114], (**d**) sensing of anti-CCP RA autoantibodies in ZnO NRs platforms with and without streptavidin [115], (**e**) anti-mouse IgG detection with ZnO NWs deposited on microfluidic channels [116], and (**f**) IgG-functionalized ZnO for cancer biomarker detection (n α-fetoprotein and carcinoembryonic antigen) [117].

While 0D ZnO nanoparticles can serve as intrinsic fluorophores, recent research has increasingly focused on 1D and 2D nanostructures, which bring about superior signal amplification and directional light control [34]. Early fluorescence-based systems primarily targeted environmental contaminants, including organic and inorganic pollutants (Figure 10a) [113]. In contrast, current efforts have shifted toward biomedical analyte detection, leveraging the near-band-edge (NBE) and deep-level emission (DLE) characteristics of ZnO to achieve precise and selective fluorescence modulation [5]. For instance, Meeseepong et al. [108] developed a biosensor based on ZnO nanorods grown on magnetic bead seeds, integrated within a microfluidic platform for the detection of cardiac troponin I (cTnI). The detection occurred through aptamer–DNA hybridization in the 50–500 pg mL^−1^ range (Figure 10b), with fluorescence intensity amplified 2.35-fold in magnetic bead–ZnO nanorods compared to control devices lacking nanorods. This configuration enabled continuous, real-time biomarker monitoring under physiological conditions.

The multiplexing capability of ZnO fluorescence systems was later demonstrated by Soundharraj et al. [118], who synthesized nitrogen-doped ZnO nanoparticles (N-ZnO NPs) for the simultaneous detection of urea, glucose, sucrose, and bovine serum albumin (BSA) (Figure 10c). The 10 nm N-ZnO particles exhibited intense emission at 385 nm, while analyte-induced selective quenching allowed for clear discrimination. Among the tested targets, urea produced the highest quenching efficiency (54%) and the lowest LOD (4.93 ± 0.02 µM), outperforming glucose (35%, 5.40 ± 0.02 µM), sucrose (33%, 7.11 ± 0.03 µM), thiourea (27%, 10.0 ± 0.01 µM), and BSA (14%, 14.7 ± 0.01 µM). Real-sample analysis using blood serum confirmed at least 90% recovery for urea concentrations ranging from 4.2 to 42 mM.

Fluorescent biosensors based on ZnO nanostructures have also shown great promise for the ultrasensitive detection of proteins, particularly in 1D configurations. Guo et al. [117] immobilized anti-bovine immunoglobulin G (IgG) on ZnO nanowires integrated into a microfluidic device, achieving a 10–20-fold fluorescence enhancement compared to planar ZnO films (Figure 10f). The system exhibited wide detection ranges: 1 pg mL^−1^ to 1 µg mL^−1^ for α-fetoprotein (AFP) and 100 fg mL^−1^ to 1 µg mL^−1^ for carcinoembryonic antigen (CEA), with corresponding LODs of 1 pg mL^−1^ and 100 fg mL^−1^, respectively. Similarly, Sang et al. [116] developed a highly sensitive platform for the detection of dye-labeled mouse IgG (417 fM–41.7 nM) by functionalizing ZnO nanowires with APTES and biotin-NHS ester (Figure 10e). The remarkable sensitivity of these 1D ZnO biosensors stems from their high surface-to-volume ratio, which enhances biomolecule immobilization and boosts fluorescence coupling efficiency.

### 4.3. Surface-Enhanced Raman Scattering (SERS) Spectroscopy

SERS-based biosensing platforms exploit nanostructured substrates to achieve highly sensitive and specific molecular detection. Their performance largely depends on the physicochemical characteristics of the substrate, which typically involves metallic NPs, nanostructured semiconductors, such as ZnO, or hybrid metal/ZnO composites. In these systems, Raman signal enhancement primarily arises from charge-transfer interactions between the analyte molecules and the ZnO surface [20,119].

Optimal SERS activity is generally observed when ZnO nanostructures are decorated with plasmonic Au or Ag NPs, creating a synergistic combination that couples electromagnetic field amplification (originating from the metallic surface plasmons), charge-transfer effects from the ZnO conduction band, and localized surface plasmon resonance (LSPR) absorption [20]. A key process underlying this enhancement is the interfacial charge-transfer transition (ICTT), in which an electron from the highest occupied molecular orbital (HOMO) of the analyte is transferred to the ZnO conduction band. The presence of metallic NPs facilitates this charge transfer by altering the polarizability of the system and promoting resonance conditions that amplify the Raman scattering signal [22,119,120].

**Figure 11:**
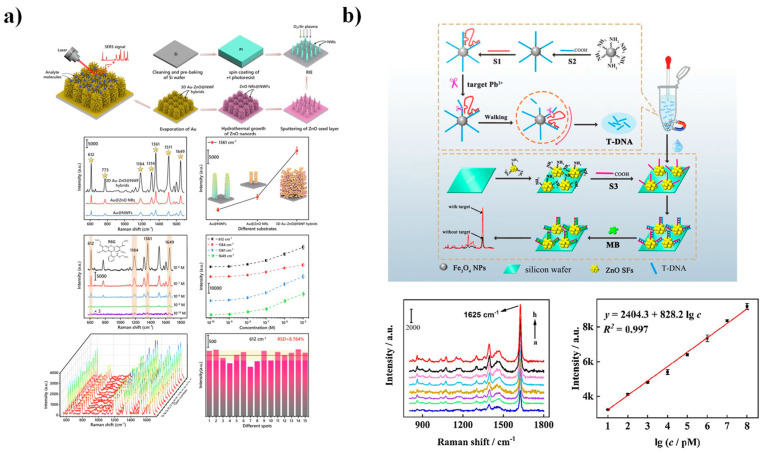
(**a**) SERS-based measurements for detection R6G on Au@NWFs, Au@ZnO NRs, and 3D Au-ZnO@NWF hybrids [8]. (**b**) Schematic diagrams of SERS biosensor for Pb^2+^ detection with Raman spectra of SERS biosensor with the increasing concentration of [pM] from 10 to 108 [123].

ZnO-based SERS substrates have enabled the ultrasensitive detection of a wide range of biomolecules, including neurotransmitters, such as serotonin, dopamine, acetylcholine, and γ-aminobutyric acid, compounds closely associated with neurodegenerative diseases like Alzheimer’s and Parkinson’s, as well as psychiatric disorders such as schizophrenia and anxiety (Figure 11a) [22,121]. For example, Deng et al. [121] developed a Ag/ZnO hybrid substrate that initially demonstrated high sensitivity toward rhodamine 6G (R6G) and was later adapted for dopamine sensing (Figure 11b). Their microcavity-inspired design achieved an exceptionally low detection limit of 5 × 10^−13^ pM for R6G, with an enhancement factor of 1.05 × 10^11^. This remarkable performance was attributed to the interplay between the piezoelectric characteristics of ZnO and optical resonances generated by whispering-gallery modes (WGMs). Similarly, a 3D nanoarchitecture composed of ZnO tetrapods decorated with AuNPs enabled the detection of amorphine, a drug used in Parkinson’s therapy, with linear response in the 100 nM–1 µM concentration range [122].

Beyond biomolecules, SERS-based biosensing has also proven highly effective for the detection of toxic heavy metals, notably Pb^2+^ a persistent contaminant known for its bioaccumulative nature and detrimental effects on renal, immune, and neurological systems. Zhang et al. [123] compared flower-like and particle-like ZnO morphologies for Pb^2+^ detection over concentrations ranging from 10 pM to 100 µM, monitoring Raman intensity at 1625 cm^−1^. The flower-like ZnO structures exhibited a markedly lower detection limit (3.55 pM), attributed to their narrower energy band, which enhanced charge-transfer efficiency and excitonic transitions, thereby amplifying the Raman signal. Selectivity assays performed in tap water revealed negligible interference from coexisting ions (Ca^2+^, Fe^3+^, Cu^2+^, Zn^2+^, Ag^+^, Al^3+^, Ni^2+^, Mg^2+^, and K^+^), underscoring the exceptional selectivity and stability of flower-like ZnO substrates toward Pb^2+^ ions.

### 4.4. Photoluminescence Spectroscopy

PL spectroscopy exploits the tunable optical properties of ZnO nanostructures for the ultrasensitive detection of biomolecules. As discussed above, these optical properties are largely governed by the synthesis route and the nature and density of structural defects. The miniaturization of ZnO into various morphologies provides a powerful strategy for engineering specific defect profiles and tailoring emission characteristics.

**Figure 12:**
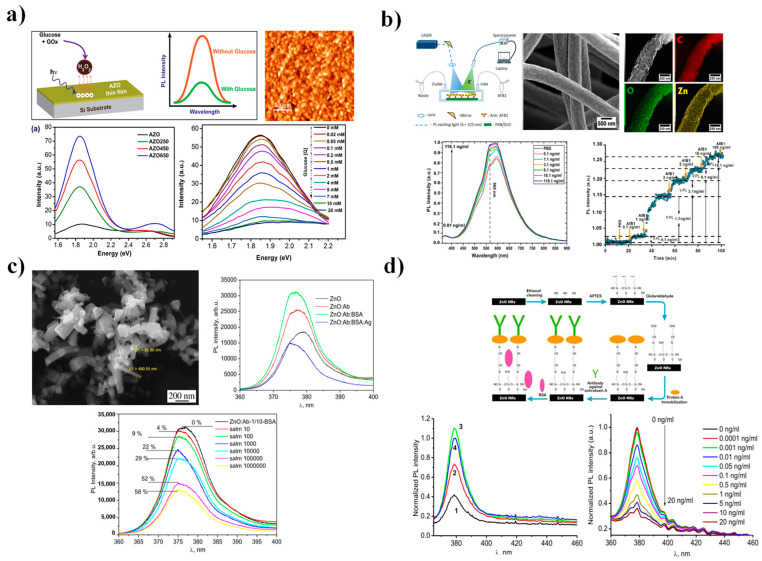
(**a**) Al-doped ZnO films as a transducer platform for glucose detection on quenching PL emission [124]; (**b**) experimental setup for PL-based AFB1 detection on PAN/ZnO20nm/APTES/GA/Anti-AFB1 structure [87]; (**c**) immobilization of anti-Salmonella antibodies ZnO nanorods following NBE emission from PL spectra to *Salmonella* Ag detection at different concentrations [125]; (**d**) Bioselective of Glass/ZnO-NRs/Protein-A/BSA&Anti-OTA structure following PL evolution spectra [7].

PL-based biosensors are capable of directly transducing biomolecular interactions, such as protein adsorption or DNA hybridization, into measurable optical responses. These responses may include intensity enhancement or quenching, spectral shifts (blue or red), the appearance or disappearance of distinct emission bands, and even real-time, label-free monitoring of biological processes (Figure 12).

A wide range of ZnO nanomorphologies have been explored for detecting diverse targets, including viruses [27], glucose [124], endocrine disruptors [7], and cells [110]. PL emission in ZnO generally consists of two components: a) a sharp near-band-edge (NBE) emission in the ultraviolet region (~388 nm), typically observed in high-crystallinity nanostructures such as 1D or 3D morphologies synthesized by vapor-phase techniques (e.g., CVD), and b) a broad deep-level emission (DLE) in the visible to near-infrared regions, characteristic of solution-processed materials (e.g., hydrothermal, spray pyrolysis, sol–gel methods). DLE arises from intrinsic defects, such as oxygen interstitials (O_i_) and zinc interstitials (Zn_i_), or from extrinsic modifications, including dopant incorporation or surface sensitization [9,81,125].

ZnO-based PL biosensors have been deliberately engineered to enhance sensitivity and selectivity toward specific biomolecular events. For example, Ghosh et al. [124] fabricated aluminum-doped ZnO thin films by sputtering, followed by thermal annealing at 250–650 °C, yielding highly oriented (002) crystalline structures. The PL spectra exhibited broad visible emissions that intensified with increasing temperature, displaying three major peaks at 1.9, 2.2, and 2.6 eV, corresponding, respectively, to deep O_i_ centers, conduction band–O_i_ transitions, and oxygen vacancies (V_o_) (Figure 12a). The films were used for glucose detection, where PL intensity decreased as glucose concentration rose from 20 µM to 20 mM. This quenching effect was attributed to the production of H_2_O_2_ during enzymatic glucose oxidation catalyzed by glucose oxidase, which consumed surface states and reduced emission.

Another major application domain is pathogen detection. Myndrul et al. [126] developed a label-free immunosensor using 1D ZnO nanostructures decorated with Au microparticles (MPs) for the detection of *Listeria monocytogenes* in the range of 10^5^–10^8^ CFU mL^−1^ (Figure 12b). The nanorod-based architecture outperformed other morphologies due to its superior crystallinity and chemical stability, achieving a limit of detection of 8.3 × 10^2^ CFU mL^−1^, well below that of conventional culture-based assays. The observed PL quenching was attributed to changes in the ZnO surface potential following antibody adsorption onto the nanorods. Similarly, Viter et al. [125] used PL spectroscopy for detecting Salmonella using ZnO nanorods (Figure 12c). Upon immobilization of anti-Salmonella antibodies, the NBE emission at 376 nm (λ_exc_ = 355 nm) progressively decreased as bacterial concentration increased from 10^2^ to 10^6^ cells mL^−1^. The quenching was associated with specific antigen–antibody interactions, which altered surface recombination dynamics and modulated carrier density.

In the context of toxin detection, ZnO nanorod-based PL platforms have been applied for identifying ochratoxin A (OTA)—a mycotoxin of significant food safety concern—across an exceptionally wide concentration range (0.0001–10 ng mL^−1^). Under 377 nm excitation, NBE-based PL measurements revealed a highly sensitive response within the 0.1–1 ng mL^−1^ interval, achieving a LOD of 0.01 ng mL^−1^, approximately one order of magnitude lower than standard ELISA assays. Furthermore, this system demonstrated excellent selectivity against structurally similar analogs, confirming its robustness for real-sample detection [7] (Figure 12d).

## 5. ZnO-Based Biosensors Targeting SARS-CoV-2: Perspectives and Future Directions

At the end of 2019, the world faced the emergence of a novel respiratory disease caused by the severe acute respiratory syndrome coronavirus 2 (SARS-CoV-2). By early 2020, COVID-19 had escalated into a global health emergency due to its high transmission rate and rapid spread [105,127,128]. In response, nations implemented a range of mitigation strategies, including lockdowns, mask mandates, social distancing, and enhanced hygiene protocols. Mass vaccination campaigns, launched soon after, brought about a substantial reduction in severe cases and mortality rates [105,129,130].

SARS-CoV-2 is an approximately 130 nm spherical virus characterized by its spike-like projections. Its genome shares roughly 80% identity with SARS-CoV and 50% with MERS-CoV. The virion comprises four major structural proteins: spike (S), envelope (E), nucleocapsid (N), and membrane (M), with the S protein—particularly its receptor-binding domain (RBD)—playing a crucial role in viral entry to cells [131,132].

**Figure 13:**
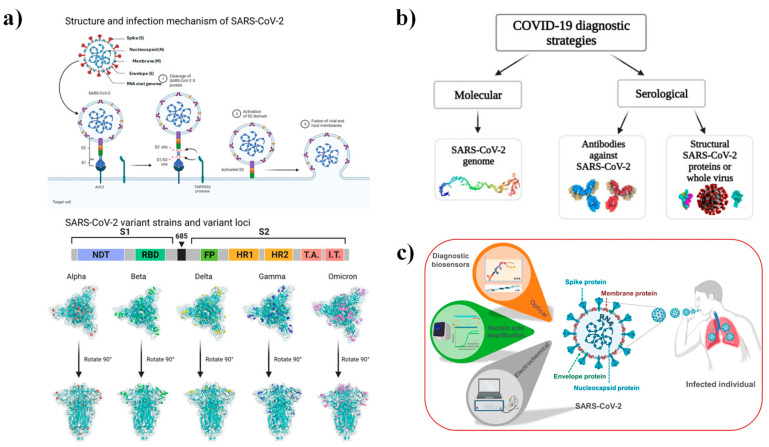
(**a**) Biology and serology of SARS-CoV-2 infection: infection mechanism, SARS-CoV-2 variants [130]; (**b**) strategies for adequate COVID-19 diagnosis [105]; (**c**) schematic representation of different sensing platforms for SARS-CoV-2 detection [132].

Early diagnostic methods, primarily molecular (reverse-transcription polymerase chain reaction, RT-PCR) and serological (Enzyme-Linked Immunosorbent Assay, ELISA) assays, offered high specificity but were hindered by long processing times, complex sample preparation, and the need for specialized instrumentation [129,132,133]. To overcome these challenges, biosensors have emerged as an appealing alternative, offering rapid, sensitive, and portable diagnostic solutions [5,105,133]. Within this framework, ZnO-based nanostructures have drawn growing attention owing to their high isoelectric point, biocompatibility, and tunable optoelectronic properties (Figure 13).

Initial developments in ZnO-based SARS-CoV-2 biosensing built upon the functionalization of ZnO nanostructures with viral antigens or antibodies. Li et al. fabricated a paper-based analytical device (PAD) using ZnO nanowires as electrodes in electrochemical impedance spectroscopy (EIS). This platform successfully detected HIV P24 antigen (0.4 pg mL^−1^) and SARS-CoV-2 IgG antibodies (CR3022) in serum, reaching a limit of detection of 10 ng mL^−1^ [134]. Likewise, Kim et al. designed a fluorescent immunoassay using ZnO nanowires deposited on a microplate, where the SARS-CoV-2 N protein directly bound to the ZnO surface without requiring APTES or GA linkers [135]. This system outperformed commercial assays, enabling early detection even in asymptomatic patients. Sportelli et al. further carried out a chemiluminescence enzyme immunoassay (CLEIA) using ZnO nanoparticles in nasopharyngeal swabs, achieving up to 90% antigen reduction and confirming the intrinsic antiviral potential of ZnO nanostructured materials [131].

Recent advances have focused on directly integrating ZnO nanostructures into biosensing devices to optimize morphology and minimize immobilization steps. Fiber-optic and microfluidic array platforms have improved detection speed and portability [105,128,132]. For example, Nunez et al. developed an impedimetric biosensor featuring ZnO nanorods functionalized with the wild-type SARS-CoV-2 spike (S) protein. The device detected anti-S monoclonal antibodies within the 200–1200 ng mL^−1^ range, achieving LOD and (limit of quantification) LOQ values of 52.55 and 159.23 ng mL^−1^, respectively—comparable to standard immunoassays [136]. In parallel, Vafabakhsh et al. [137] introduced a colorimetric aptasensor based on a chitosan/ZnO/carbon nanotube (ChF/ZnO/CNT) hybrid functionalized with a COVID-19-specific aptamer. The peroxidase-like catalytic activity of this system enabled detection across 1–500 pg mL^−1^, with a LOD of 0.05 pg mL^−1^. Similarly, Nunez et al. [138] developed a voltammetric immunosensor using ZnO nanorods functionalized with epitopes from various SARS-CoV-2 variants. The platform reached LOD values of 0.14, 0.19, and 0.35 ng mL^−1^ for the wild-type, Gamma, and Omicron variants, respectively. These sensors were validated using human serum samples, confirming their adaptability to emerging variants and suggesting applicability to future viral pathogens.

**Figure 14:**
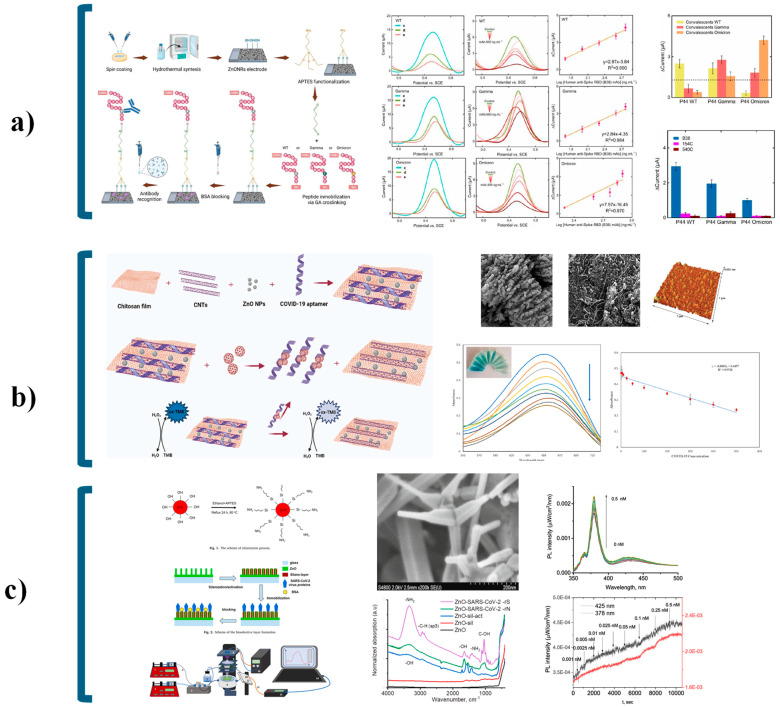
(**a**) Assembly process of the P44 peptide-based electrochemical immunosensor (ZnO NRs on FTO); electrochemical performance of P44 peptide-based immunosensors for WT, Gamma, and Omicron; validation of P44 peptide-based immunosensors and correlation with neutralization titers; selectivity using pooled convalescent sera from WT and Gamma [138]. (**b**) Schematic representation and characterization of the platform for the detection of COVID-19; FESEM analysis of synthesized ZnO NPs and ChF/ZnO/CNT nanohybrid; AFM image of ChF/ZnO/CNT nanohybrid [137]. (**c**) Optical setup featuring a microfluidic system showcasing evidence of ZnO tetrapods functionalization (FTIR); immobilization with recombinant proteins (rS and rN), and PL measurements. This includes kinetic assessments of the ZnO/SARS-CoV-rS nanostructures about anti-SARS-CoV-2 antibodies [27].

Optical biosensing strategies have also gained momentum. Viter et al. employed ZnO nanotetrapods functionalized with recombinant spike (rS) and nucleocapsid (rN) proteins to detect SARS-CoV-2 antibodies through photoluminescence quenching. The large surface area and stable PL emission of ZnO tetrapods yielded a LOD of 0.01 nM. Moreover, integrating microfluidic channels with room-temperature PL detection reduced the total assay time to 15–20 min [27] (Figure 14).

Collectively, these innovations underscore the versatility of ZnO nanostructures for developing rapid, accurate, and cost-effective SARS-CoV-2 biosensors. Their inherent adaptability, combined with tunable optical and electronic properties, positions ZnO as a cornerstone material for next-generation diagnostic platforms—capable of evolving alongside emerging pathogens and public health demands.

**Figure 15:**
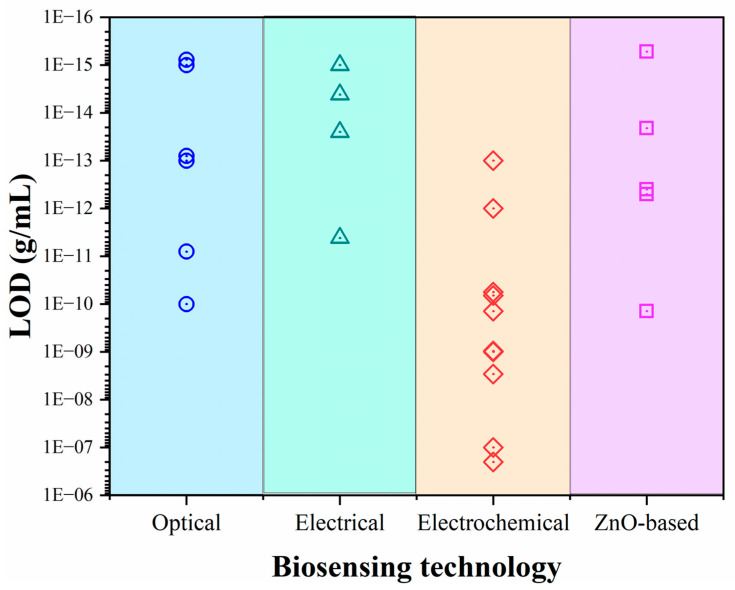
Comparison of limits of detection (LOD) values for various biosensing technologies that use nanostructured platforms in the detection of SARS-CoV-2-related agents (N or S proteins, IgM or IgG antibodies). Data extracted from [137,138,139,140].

Figure 15 provides a comparative overview of limit of detection values reported for various biosensing technologies targeting SARS-CoV-2-related biomarkers—including N and S proteins, IgM/IgG antibodies, and viral particles—grouped according to their transduction mechanisms: optical, electrical (e.g., FET-based), electrochemical, and ZnO-based platforms [139,140]. Among these, optical and FET-based sensors exhibit the highest sensitivities (in the pg mL^−1^ to fg mL^−1^ range) and fastest response times, often reaching real-time detection. ZnO-based devices stand out as a cost-effective and versatile alternative, offering ultrasensitive performance while maintaining structural tunability, surface functionalization flexibility, and compatibility with scalable fabrication.

## 6. Future Directions

Upcoming developments in ZnO-based biosensors will focus on enhancing sensitivity, selectivity, portability, and real-time monitoring. Special attention will be paid to selecting the appropriate ZnO nanomorphology, ranging from 0D to 3D, based on the specific application. This selection must align with the synthesis methodology (physical or wet-chemical) and its parameter optimization to fine-tune the optoelectronic and surface properties of ZnO.

- 0D nanostructures provide label-free detection via quantum confinement and strong fluorescence signals. Their integration with metal NPs further enhances optical responses and lowers the limit of detection.
- 1D nanostructures offer high surface areas for dense biomolecule immobilization and efficient charge transport, enhancing sensitivity and reducing signal noise. Techniques like hydrothermal synthesis, CVD, or template-assisted growth enable their integration onto various substrates with controlled orientation and density.
- 2D nanostructures enable uniform biomolecule coverage and superior electron mobility, often forming hybrid heterostructures with synergistic properties.
- 3D morphologies provide ultra-high surface areas, ideal for multiplexed detection and rapid response times, often within minutes or in real-time.

Progress in this field requires multidisciplinary and transdisciplinary collaboration. Experts in materials science, nanotechnology, biology, chemistry, and engineering must work together to develop clinically validated, reproducible platforms. Key challenges include ensuring long-term device stability and standardizing biomolecule immobilization protocols (e.g., silanization, covalent crosslinking). Addressing these will pave the way for ZnO-based biosensors to become practical tools for affordable, rapid, and multiplexed diagnostics in global healthcare.

## 7. Conclusions

This review systematically examines the impact of ZnO nanomorphology and synthesis-related parameters on biosensor performance. Two main aspects are emphasized: (1) the choice of dimensionality and synthesis method as critical factors influencing sensitivity, selectivity, and device functionality, and (2) the need to tailor optoelectronic properties for specific biosensing applications.

The selection of a synthesis method—either physical or wet-chemical—determines key characteristics of ZnO nanostructures, including morphology, crystallinity, and surface chemistry. Physical approaches typically yield highly crystalline nanomaterials with minimal structural defects, offering efficient electron transport. In contrast, wet-chemical methods allow the fabrication of diverse morphologies (e.g., nanoparticles, nanorods, nanoflowers) at lower temperatures, making them suitable for scalable processes and integration with flexible substrates.

From an application standpoint, the integration of ZnO nanostructures across various dimensionalities (0D to 3D) significantly influences biosensor sensitivity, selectivity, and operational stability. While 0D and 3D forms provide abundant active sites for biomolecule immobilization and signal amplification, 1D and 2D morphologies offer enhanced charge transport and large functional surfaces. The morphological versatility of ZnO also allows effective conjugation with diverse bioreceptors (e.g., antibodies, enzymes, aptamers), enabling its application in the detection of a broad range of biological targets, including emerging pathogens.

Morphology-driven signal amplification enables ultra-sensitive, label-free detection of biomolecules, even at trace levels. This versatility positions ZnO as a promising candidate for next-generation optical biosensors, particularly in compact and point-of-care diagnostic platforms. Recent progress in ZnO nanotechnology has not only improved detection limits, response times, and operational stability, but also facilitated the rapid reconfiguration of sensing platforms to respond to emergent health threats such as SARS-CoV-2.

## Figures and Tables

**Table 1:** Summary of advantages, disadvantages, and typical application areas of different biomolecule immobilization strategies used in biosensing.

Strategy	Advantages	Disadvantages	Sensing Application Areas	Ref.
Covalent attachment	Strong and long-lasting binding High stability under harsh conditions. Minimize non-specific adsorption.	Irreversible immobilization. Complex optimization, dependent on temperature, pH, and reaction time. Risk of biomolecule denaturation.	Long-term biosensors (e.g., implantable). Optical biosensors.	[26,35,78]
Physical adsorption	Simple and rapid process, no need for reducing agents or modified molecules Preserves biomolecule conformation. Suitable for a wide range of biomolecules.	Weak binding (sensitive to ionic strength). Possible non-specific adsorption, which may affect sensor specificity. Low reproducibility.	Short-term or disposable sensors. Protein and antibody sensors.	[35]
Entrapment/encapsulation	Protects biomolecules from harsh environments. In porous ZnO (e.g., nanotubes), biomolecule immobilization density is maximized.	Slow analyte diffusion due to dense matrices. Requires optimization of polymer-ZnO ratios. Reduced signal-to-noise ratio.	Enzymatic sensors (e.g., cholesterol).	[79]
Cross-linking	Facilitate multi-enzyme system stability. Strong attachment and reduced leaching. Allows biomolecule orientation for accessibility.	Blocks active sites. Risk of aggregation.	Multi-analyte detection systems.	[79]

**Table 2:** Summary of ZnO nanostructures used as transducer platforms in various biomolecule immobilization strategies and their corresponding biosensing performance.

ZnO Classification	Nanoarchitecture Configuration	Target Analyte	Bioselective Layer	Linear Range	Limit of Detection (LOD)	Sensitivity	Immobilization Strategy	Biosensing Application	Ref.
0D	APTES-capped ZnO QDs	Dopamine	NM	0.05–10 µM	12 nM	NM	Physical adsorption	Optical biosensor (Fluorescent mechanism)	[36]
	Si/CA 19-9 Ab/CA 19-9/ZnO QDs	antigen CA19-9	CA 19-9 Ab	0.1–180 U mL^−1^ 1–180 U mL^−1^	0.04 U mL^−1^ 0.25 U mL^−1^	0.47 µA/U ml^−1^	Covalent bonding	Electrochemical mechanism Optical mechanism	[71]
1D	GaN/ZnO NRs/β-D- glucose	Glucose	Glucose oxidase	0.5–30 mM	0.5–30 mM	1.4%/mM	Covalent bonding	Optical sensing	[72]
	Au/ZnO NRs	Phenol	Tyrosinase	>20 µM <20 µM	0.623 µM	103.08 µA mM^−1^ 40.76 µA mM^−1^	Physical adsorption	Optical sensing	[73]
	Ag-ZnO NRs-mAb-E2	17β-Estradiol (E2)	Monoclonal antibody of E2 (mAb-E2)	0.1–200 pg mL^−1^	0.01 pg mL^−1^	0.01 pg mL^−1^	Covalent bonding	Electrochemical sensing	[37]
	ZnO NRs on Pt film	Glucose	Glucose oxidase	0.5–3 mM (physical adsorption) 0.5–8.5 mM (cross-linking)	NM	17.7 mA cm^−2^ M^−1^	Physical adsorption and cross-linking	Electrochemical sensing	[81]
	Whatman filter/ZnO NRs	Glucose (GO) and uric acid (UA)	Glucose oxidase uricase	0.01–10 mM (GO) 0.01–5 mM (UA)	3 µM (GO) 4 µM (UA)	NM	Physical adsorption	Colorimetric method	[82]
	Ag/ZnO NRs	Dextrose	Glucose oxidase	0–10 mM	0.012 mM	26.29 nm mM^−1^	Physical adsorption	Optical sensing	[83]
	Glass/ZnO NRs	Human leukemic cells B-lymphoblastoid cell line IM9	Anti-CD19-FITC*	10 to 500 cells	NM	10 till 1000 cells per mm^2^	Physical adsorption	Optical sensing	[84]
	Neutral Red-nanostructured ZnO (NR-AZO)	Nicotinamide adenine dinucleotide (NADH)	Lactate dehydrogenase (LDH)	0.1–1 mM	22 µM	0.45 µA cm^−2^	Physical adsorption	Electrochemical sensing	[85]
	Au hybridized ZnO NRs	HPV-16 DNA	Thiolated ssDNA (HPV-16 E6)	10^−7^–10^−15^ M	1 fM	1 fM	Covalent bonding	Electrochemical biosensor	[86]
	polyacrylonitrile/ZnO (PAN/ZnO) nanofibers	Aflatoxin B1 (AFB1)	anti-AFB1	0.1–100 ng mL^−1^	39 pg mL^−1^	0.1–20 ng ml^−1^	Covalent bonding	Optical biosensor	[87]
	ZnO-NRs	Ochratoxin A (OTA)	Anti-OTA	10^−4^–20 ng mL^−1^	0.0001 ng mL^−1^	0.1–1 ng ml^−1^	Covalent bonding	Immunosensor	[7]
2D	Si/ZnO film	Grapevine virus A (GVA)-type	Anti-GVA	1 pg mL^−1^ to 1 µg mL^−1^	NM	1–10 ng ml^−1^	Physical adsorption	Optical sensing	[88]
	ZnO films	DNA of the dengue virus	specific probe strand	NM	NM	1 × 10^−15^ moles of DNA	Physical adsorption	Optical sensing	[74]
	Glass/Au/ZnO thin film/ssDNA	*Neisseria meningitidis* DNA	DNA strands	10–180 ng µL^−1^	5 ng µL^−1^	0.03°/(ng µL^−1^)	Physical adsorption	Optical sensing	[75]
3D	ZnO/Si nanoneedles/AgNPs	Rhodamine 6G (R6G)	NA	10^−6^ –10^−12^ M	1.1 × 10^−16^ M	NM	Physical adsorption	Optical sensing	[55]
	Bare and Nafion-modified ZnO pyramids	Glucose	Glucose oxidase	0.05–8.2 mM	0.01 mM	NM	Physical adsorption	Electrochemical sensing	[89]
	ZnO/DPGP/ADH ZnO/DPGP/GO_x_	Ethanol Glucose	Alcohol dehydrogenase Glucose oxidase	10–650 µM 1–50 mM	2.1 µM 3.6 µM	7.6 µA cm^−2^ mM^−1^ 53.9 µA cm^−2^ mM^−1^	Covalent bonding	Electrochemical sensing	[90]
	ZnO/SARS-CoV-rS ZnO/SARS-CoV-rN (tetrapods)	SARS-CoV-2 proteins	SARS-CoV-2 spike/nucleocapsid proteins	0.025–0.5 nM 0.3–1 nM	0.01 nM 0.3 nM	NM	Covalent bonding (cross-linker)	Immunosensor	[91]
	PCN-224/nano-zinc oxide nanocomposite	Human papillomavirus (HPV-16) DNA	Thiolated Capture DNA + DNA-dopamine conjugates	1 fM–1 nM	0.13 fM	NM	Covalent bonding	Electrooptical biosensor	[92]

Note: NM = not mentioned; NRs = nanorods; NWs = nanowires; QDs = quantum dots. Units presented in linear range, LOD and sensitivity correspond to those used in the original publications.

**Table 3:** Summary of key optical biosensing techniques based on ZnO platforms, including fundamental principles and application areas.

Optical Technique	Working Principle	Biosensing Application	Ref.
SPR/LSPR	Refractive index changes at metal interfaces	Label-free protein detection	[83,107]
Fluorescence	Emission from fluorophore-labeled analytes	High-specificity cellular imaging	[74,108]
Raman Spectroscopy	Molecular vibrational mode analysis	Pathogen identification via SERS	[55,109]
PL	NBE and DLE emissions (shift, intensity, quenching)	Label-free analyte detection with high sensitivity	[9,27,110]

## Data Availability

No new data were created or analyzed in this study. Data sharing is not applicable to this article.
